# Policy and Practice Implications of Outcome Measures of Nonpharmacological Interventions

**DOI:** 10.1093/geroni/igaf122.1726

**Published:** 2025-12-31

**Authors:** Kalisha Bonds Johnson, Daniel Siconolfi, Heather Menne, Nicole Batsch, William Zagorski, Kate Perepezko, Julie Robison, Regina Shih

**Affiliations:** Emory University, Atlanta, Georgia, United States; RAND Corporation, Santa Monica, California, United States; Miami University, Oxford, Ohio, United States; Consultant, Florida, United States; American Senior Care Centers, Inc., Nashville, Tennessee, United States; Miami University, Oxford, Ohio, United States; University of Connecticut, Farmington, Connecticut, United States; Emory University Rollins School of Public Health, Atlanta, Georgia, United States

## Abstract

Nonpharmacological interventions often measure the wellbeing of persons living with dementia (PLWD) and their family care partners by assessing their quality of life (QOL). These interventions use various outcome measures to evaluate and assess improvement in the QOL of PLWD and their care partners. Quality of life has been operationalized as a multidimensional concept, which makes comparing outcome measures across interventions challenging. In this presentation, we provide an in-depth examination of the outcome measures used in 37 nonpharmacological interventions in a resource identified by the National Alzheimer’s and Dementia Resource Center at the request of the Administration for Community Living (ACL), which includes both evidence-based and evidence-informed interventions based on the ACL requirements. We organized the outcome measures from these 37 interventions into four main sectors identified by Lawton’s multidimensional concept of QOL —behavioral competence (e.g. activities of daily living and cognitive functioning), perceived QOL (e.g. relationships and health), objective environment (e.g. neighborhood characteristics and social networks), and psychosocial wellbeing (e.g. emotions and mental health). We also highlight who the outcome measures were about as well as who completed the measures in each intervention (e.g., the person living with dementia, the care partner, or both). Findings provide opportunities to showcase how focusing on a more multidimensional approach to measuring the impact of interventions on QOL may reveal challenges and policy ramifications that influence one or both members of the dementia dyad.

